# Needs and Perspectives on Upper Limb Prostheses Among Children and Adolescents With Upper Limb Differences

**DOI:** 10.1001/jamanetworkopen.2026.20122

**Published:** 2026-06-25

**Authors:** Kevin Wendo, Crystal Chigbu, Obafemi Ekundayo, Oluwaseun Ayodeji, Fiyinfoluwa Ayoola, Adebowale Adekemisola Ayoola, Gbolamide Ogunkua, Vindication Joseph, Gift Anyanwu, Séverine Guisset, Jade Ward, Benoît Herman, Stéphane Moniotte, Raphael Olszewski

**Affiliations:** 1 Université Catholique de Louvain (UCLouvain), Cliniques Universitaires Saint-Luc, Department of Pediatrics, Brussels, Belgium; 2 UCLouvain, Institut de Recherche Expérimentale et Clinique (IREC), Neuro Musculo Skeletal (NMSK) Lab, Brussels, Belgium; 3 UCLouvain, IREC, NMSK Lab, Oral and Maxillofacial Surgery (OMFS) Lab, Brussels, Belgium; 4 The IREDE Foundation, Lagos, Nigeria; 5 Slum and Rural Health Initiatives, Oyo, Nigeria; 6 VMO Aero, Lagos, Nigeria; 7 The Civic Centre, Lagos, Nigeria; 8 AidtoAid International, Lagos, Nigeria; 9 Forvis Mazars, Lagos, Nigeria; 10 UCLouvain, Louvain Institute of Data Analysis and Modeling in Economics and Statistics (LIDAM), Statistical Methodology and Computing Support (SMCS), Louvain-la-Neuve, Belgium; 11 UCLouvain, Institute of Health and Society (IRSS), Brussels, Belgium; 12 AMPrint Center, Rochester Institute of Technology, Rochester, New York; 13 UCLouvain, Institute of Mechanics, Materials and Civil Engineering, Louvain-la-Neuve, Belgium; 14 UCLouvain, Louvain Bionics, Louvain-la-Neuve, Belgium; 15 UCLouvain, Cliniques Universitaires Saint-Luc, Department of Oral and Maxillofacial Surgery, Brussels, Belgium; 16 Faculty of Medicine, Lazarski University, Warsaw, Poland

## Abstract

**Importance:**

In low- and middle-income countries (LMICs), limited access to adequate prosthetic care hinders the psycho-socio-motor development and educational progress of children and adolescents with upper limb differences. The suitability of current pediatric prosthetic solutions and the needs and expectations of children and adolescents with upper limb differences regarding upper limb prostheses (ULPs) remain understudied in LMICs.

**Objectives:**

To examine and characterize the needs, expectations, and perceptions of Nigerian children and adolescents with upper limb differences regarding ULPs, assessing contextualized specifications for prosthetic development.

**Design, Setting, and Participants:**

This qualitative study was conducted from July 20 to July 23, 2024, in Lagos, Nigeria, among 25 children and adolescents with upper limb differences purposively selected from The IREDE Foundation prosthetic care program; recipients were aged 5 through 20 years who possessed at least 1 ULP. This study involved semistructured interviews and focus groups of Nigerian children and adolescents with upper limb differences. Transcripts were analyzed using thematic analysis between August 2024 and May 2025.

**Main Outcomes and Measures:**

The needs and perceptions of children and adolescents with upper limb differences regarding ULPs.

**Results:**

A total of 25 children and adolescents with upper limb differences (mean [SD] age, 13.5 [4.2] years; 15 male [60%]) enrolled in the study; 14 were interviewed, and 11 engaged in focus groups. The predominant cause of upper limb differences was traumatic amputations (16 [64%]), and the predominant level of upper limb differences was transhumeral (16 [64%]). Seven themes were identified from the interviews and focus groups. First, the children and adolescents with upper limb differences described the technical specifications of an ideal ULP by (1) highlighting the functionalities and task performance it should enable; (2) expressing their need for an active ULP and the characteristics of effective terminal devices; (3) specifying user-defined requirements for quality of prostheses components; (4) describing factors contributing to comfortable wear and use; and (5) explaining the necessity for anthropomorphism (shape and color) of ULP designs. Second, the psychosocial consequences of the conditions of the children and adolescents with upper limb differences and ULP use emerged: adverse repercussions (6) on self-concept and (7) in interactions with their social network, which were major factors in ULP acceptance. Overall, children and adolescents with upper limb differences expressed the need for an active, anthropomorphic, sturdy ULP, enabling them to actively engage with their peers.

**Conclusions and Relevance:**

In this qualitative study, children and adolescents with upper limb differences expressed perceptions regarding their current ULP and described the requirements of prosthetic devices meeting the psychosocial and occupational needs within their socialcultural environment. Future studies could incorporate these findings into the development of environment-specific pediatric ULPs.

## Key Points

**Question:**

What do children and adolescents with upper limb differences from low- and middle-income countries need and expect from upper limb prostheses (ULPs)?

**Findings:**

This qualitative study of 25 Nigerian children and adolescents with upper limb differences found that participants specified an ideal ULP as active, anthropomorphic, and robust enough to facilitate their independence and their confidence in social engagement. For children and adolescents with upper limb differences, the main psychosocial repercussions of their conditions and ULP use were impaired self-concept, stigmatization, and discrimination.

**Meaning:**

These findings suggest that children and adolescents with upper limb differences from low- and middle-income countries require context-specific prosthetic solutions to meet their functional, psychosocial, and quality-of-life needs.

## Teaser

This qualitative study examines and characterizes the needs, expectations, and perceptions of Nigerian children and adolescents with upper limb differences regarding upper limb prostheses within their sociocultural and daily environments.

## Introduction

Upper limb differences (ULDs), defined as the absence or malformation of an upper extremity segment, may be congenital or acquired (eg, trauma or disease).^1,2,3,4^ During childhood development, ULDs can result in physical (eg, overuse syndromes) and psychosocial challenges (eg, impaired self-concept).^2,3,4^ To mitigate these effects, upper limb prostheses (ULPs) may be provided to restore appearance and other function.^1,3,4^ Evidence supports a pediatric user–centered approach to prosthetic design to address children’s needs and preferences adequately.^3,4,5,6^ For example, children with congenital below-elbow differences show high and increasing rates of prosthesis rejection,^5,6,7,8^ partly because many retain near-typical function without a device, have no sense of loss, manifest improved self-acceptance during adolescence, and then use prostheses for social or cosmetic purposes.^4,8,9,10,11^ Activity-specific prostheses offering functional benefits are therefore preferred.^8,12^ Consequently, assessing end-user preferences is essential to determine the optimal balance between user needs, available technological options, and structural constraints (eg, cost and health care accessibility).^4,8,9,11,12,13,14^

Most children with ULDs reside in low- and middle-income countries (LMICs), where access to prosthetic and rehabilitation services is limited and epidemiologic data are scarce.^15,16,17,18,19,20,21,22^ Limited study of the functional and psychosocial needs in this population hampers the design of context-appropriate prosthetic solutions.^15,17,18,21,23^ The limited studies that do exist emphasize functionality, affordability, and durability as key factors associated with ULP use.^24^ However, existing research is largely quantitative, focused on congenital ULDs, and conducted in high-income countries (HICs), thereby limiting transferability.^6,7,9,10,25^ Consequently, pediatric prosthetic solutions in LMICs are often based on data generated in HICs and may not suit the environmental and structural constraints in LMICs.^17,18^

Therefore, in-depth qualitative research is needed to explore lived experiences and priorities of children and adolescents with ULDs in LMICs. This study aims to investigate the needs, perceptions, and expectations of children and adolescents with ULDs living in the lower-middle–income country of Nigeria regarding ULP use within their sociocultural and daily environment.

## Methods

### Study Design

We conducted a qualitative phenomenological study among Nigerian children and adolescents with ULDs to explore their lived experiences and perceptions. An inductive thematic approach using semistructured focus group discussions and interviews was used. Participants provided written assent, and caregivers provided written consent. This study received ethical approval from the Lagos State Health Research Ethics Committee and followed the Standards for Reporting Qualitative Research (SRQR) reporting guideline.^26^

### Participants

Purposeful sampling was used to recruit children and adolescents with ULDs enrolled in the ULP program of The IREDE Foundation (TIF), a Nigerian nonprofit providing pediatric prosthetic services. Eligibility criteria included (1) ULD diagnosis, (2) age 5 to 18 years (with inclusion of young adults enrolled as minors), and (3) participation in TIF program. Exclusion criteria were inability to communicate verbally and isolated lower-limb amputation. Of 45 eligible participants contacted, 25 consented to participate. Sex representation was not predetermined to maximize recruitment.

### Data Collection

Data were collected at TIF headquarters in Lagos, Nigeria, between July 20 and July 23, 2024, using 3 modalities to maximize access (Table 1) and enhance methodological quality: in-person focus group, online focus group, and individual telephone interviews. Due to unforeseen logistical reasons (eg, transportation or internet connectivity), the 2 in-person focus groups included 6 participants (age range, 10-17 years) and 3 participants (age range, 8-10 years), respectively; the online focus group included 2 children and adolescents with ULDs (aged 18 years); and 14 other participants were interviewed. Each participant contributed through only 1 modality. The online focus group was conducted via videoconference.

**Table 1.** Characteristics of Participants

Characteristic	Participants, No. (%) (N = 25)
Age, y	
6-9	6 (24)
10-12	4 (16)
13-15	6 (24)
16-18	6 (24)
19-20	3 (12)
Age, mean (SD), y	13.5 (4.2)
Sex	
Female	10 (40)
Male	15 (60)
Etiology of ULD	
Trauma	16 (64)
Congenital	6 (24)
Sickness	2 (8)
Mismanagement by traditional bone healers	1 (4)
ULD level	
Transradial	9 (36)
Transhumeral	16 (64)
Side of body of ULD	
Left	10 (40)
Right	15 (60)
Age at first prosthesis, y	
3-5	4 (16)
6-9	5 (20)
10-12	2 (8)
13-15	11 (44)
16-18	2 (8)
19-20	1 (4)
Age at first prosthesis, mean (SD), y	11.4 (4.5)
No. of prostheses used	
1	20 (80)
2	4 (16)
3	1 (4)
Type of prostheses used	
Active	0
Passive	23 (92)
Passive with ITDs	2 (8)
Perception of ULP usefulness	
Very useful	9 (36)
Useful	10 (40)
Not useful	4 (16)
Not useful at all	2 (8)
Geographical distribution of country states (rural or urban)	
Abuja (urban)	1 (4)
Anambra (urban)	1 (4)
Delta (urban)	1 (4)
Edo (rural)	1 (4)
Enugu (rural)	1 (4)
Kwara (rural)	1 (4)
Lagos (urban)	11 (44)
Ogun (rural)	3 (12)
Osun (rural)	1 (4)
Oyo (rural)	4 (16)

Abbreviations: ITDs, interchangeable terminal devices; ULD, upper limb difference; ULP, upper limb prosthesis.

Trained TIF staff (O.E., F.A., and A.A.A.; TIF Program associates) moderated discussions to foster a supportive and comfortable environment. The lead investigator (K.W.; physician) had no relationship with participants and ensured consistency across modalities. Sessions were conducted in English, with 5 interviews conducted in Yoruba for participants’ convenience and translated into English (O.E.). Field notes were collected (K.W., O.E., A.A.A, and F.A.) throughout. The mean duration of the focus groups was 70 minutes, and the mean duration of interviews was 18 minutes. Interview durations were constrained by young participants’ limited attention span and school commitments.

A semistructured interviewing guide (eMethods in Supplement 1), adapted from Sims et al,^12^ and expanded to include terminal device (TD) preferences (with illustrating photographs), was used across all modalities. Demographic (age and sex) and clinical (eg, ULD level and etiology) data were self-reported, and caregivers scored perceived ULP usefulness using personal record forms (Table 1). These demographics were reported descriptively. No financial incentives were offered, but participation certificates and logistical support were provided.

### Statistical Analysis

Transcripts were analyzed using thematic analysis between August 2024 and May 2025. Focus groups and interviews were audio-recorded, transcribed, and pseudonymized. Data were analyzed using reflexive thematic analysis as delineated by Braune et al,^27,28^ a systematic, inductive, and iterative method to identify patterns within qualitative data. This approach consists of data familiarization, open coding, pattern analysis, and categorization of codes into themes. Focus group data were coded prior to interviews. Inductive thematic saturation was reached during interview coding. All interviews were coded for confirmation. Themes and subthemes were developed iteratively and confirmed through a team process involving consensus among clinicians, prosthetics specialists, researchers, and TIF staff (K.W., C.C., O.E, O.A., G.O., V.J., G.A., S.G., J.W., and S.M.). Disagreements were resolved through discussions.

Methodological rigor was ensured through triangulation of data collection methods, “thick description” (ie, rich contextual details) of our study protocol for facilitated transferability,^29,30^ and iterative confrontation of codes and (sub)themes with the dataset. Throughout analysis, reflexive journaling and team discussions allowed us to address potential bias. Bias risk observed during data collection was documented and discussed within the research team.

## Results

### Sample Characteristics

In total, 25 children and adolescents with ULDs (mean [SD] age, 13.5 [4.2] years; 15 male [60%] and 10 female [40%]) enrolled in this study (Table 1). The predominant ULD level was transhumeral (16 [64%]), and the predominant cause was traumatic amputations (16 [64%]). All participants used a passive ULP, and 2 children and adolescents with ULDs had interchangeable TDs. Most participants (20 [80%]) were wearing their first ULP, acquired within 18 months prior to the study (17 of 25 [68%]). Most parents deemed their children’s prostheses useful (19 of 25 [76%]), but on analysis of qualitative comments, few were satisfied, desiring an active ULP and component improvements.

### Findings

Two overarching themes emerged: (1) functional and mechanical characteristics of an ideal ULP and (2) psychosocial implications. Within these overarching themes, 7 major themes were identified: (1) functionality as a prerequisite, (2) ideal active prosthetic design specifications, (3) prosthesis component quality, (4) physical comfort as key to prosthesis acceptance, (5) anthropomorphic prostheses as tools for self-concept improvement and social integration (overarching theme 1), and (6) self-perception and (7) peers’ and strangers’ attitudes (overarching theme 2) (Table 2). Thematic associations are illustrated in the Figure, with representative quotations presented in Table 3 and Table 4. Findings demonstrate that the characteristics of a physical prosthesis are inseparable from children and adolescents’ mental representations of their devices.

**Table 2.** Themes and Subthemes

Overarching and major themes	First-degree subthemes	Second-degree subthemes
Functional and mechanical characteristics of an ideal upper limb prosthesis		
Functionality as a prerequisite	Frustrating passive prosthesis	No second-degree subthemes developed
	Required functionalities for improved autonomy	Self-care; Household chores; Interpersonal interactions; Other functional abilities
Ideal active prosthetic design specifications	No subthemes developed	
Prosthesis component quality	No subthemes developed	
Physical comfort as key to prosthesis acceptance	Inadequate fitting; Excessive weight; Ineffective suspension; Insufficient prosthesis ventilation	No second-degree subthemes developed
Anthropomorphic prostheses as tools for self-concept improvement and social integration	Anthropomorphic appearance; Prosthesis color	No second-degree subthemes developed
Psychosocial implications		
Self-perception	No subthemes developed	
Peers’ and strangers’ attitudes	No subthemes developed	

**Figure.**
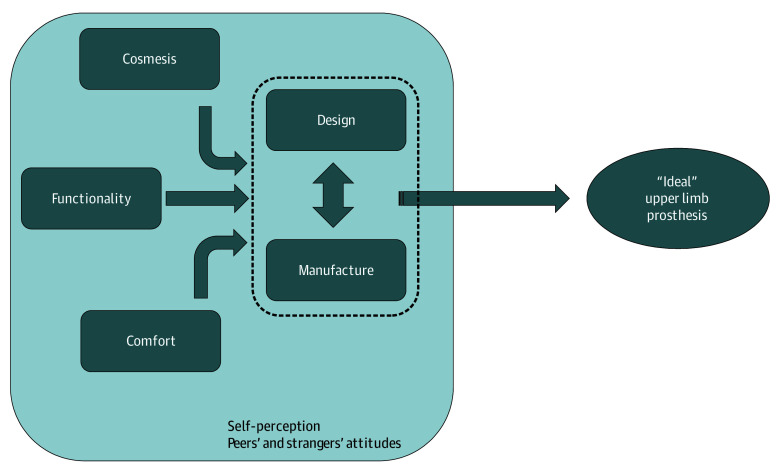
Thematic Map Representing a Conceptual Model of Pediatric Upper Limb Prostheses Acceptance in Low- and Middle-Income Countries Upper limb prostheses’ functionality, comfort, and cosmesis influence both design and manufacture stages, which are closely interrelated. An “ideal” upper limb prosthesis solution may emerge from these latter steps, after the integration of all specifications, constraints, and demands within recipients’ unique psychosociocultural context.

**Table 3.** Overarching Theme 1: Functional and Mechanical Characteristics of an Ideal Upper Limb Prosthesis

Theme and subtheme	Quotations (data collection method–sex [M/F]–age [years]–ULD level–ULD etiology)
Theme 1: functionality as a prerequisite	
Frustrating passive prosthesis	“I don’t like anything… it because it’s not functioning.” (II-M-18-TR-T)
	“… I wish I could use it to do something.” (OF-F-18-TR-T)
	“I don’t do anything. I don’t use it to do anything. I use it only… it gives me fitting [for aesthetics].” (II-M-18-TR-T)
Required functionalities for improved autonomy	
Self-care	“I’ll use it [ideal prosthesis]… to comb my hair… I’ll use it [ideal prosthesis] to cream [apply body lotion].” (PF-F-8-TR-S)
Household chores	“[I’d use it] (ideal prosthesis) to sweep, to wash, to fetch, to clean, to do any sort of things… domestic stuff.” (II-M-15-TR-C)
	“At home, I would like to be using it to be washing clothes.” (PF-F-14-TH-T)
	“I would like it to help me to wash my clothes so that… If it’s possible, I would like it.” (II-M-18-TR-S)
Interpersonal interactions	IR: “… [And] with your friends?” PT: “Shake… shake hands, hug something like that.” (TT-M-19-TH-T)
	“Play sports, football, basketball… to shake them, to be able to touch them… To shake, yeah. Shake, touch them I do not…” (II-M-15-TR-C)
	“To shake hands, play with friends.” (II-F-14-TH-T)
Other functional abilities	PT: “Swimming.” IR: “You love swimming?” PT: “I want to learn.” (II-F-14-TH-T)
	“… Let’s just say skip. I’d like to know the feeling of skipping, skipping a rope.” (OF-F-18-TR-T)
	“At school, I am a pharmacy student, I work in the lab. So, I want to use it to hold sometimes test tubes…” (II-M-19-TR-T)
	“I cannot use it to lift things… like when I want to take my food. …If I want to transport it let’s say… First, I would have first to carry the food. Then, come back for [a] cup of water. I would like to carry the two of them together.” (PF-M-16-TH-S)^a^
Theme 2: ideal active prosthetic design specifications	“If I had to build this myself… I feel like… Making it move. I’ll be able to bend the hand… Yes… I’ll make it move… I’ll be able to make it move.” (OF-F-18-TH-T)
	“The hand should be working.” (II-F-9-TR-C)
	“I should be able to control like the fingers, you can move it, or you can control fingers or even shake hands.” (II-M-15-TR-C)
	“It should be movable, and it should be flexible.” (II-F-14-TH-T)
	PT: “I want the one [terminal device] that can do everything.” IR: “Why?” PT: “So, I don’t need to be changing it.” (II-F-6-TH-A)
	“I prefer the one [terminal device] that is just there, [that] I don’t need to remove.” (OF-F-18-TH-T)
	“I’ll try the one [terminal device] with different tools that I can remove and put back, to write, to sweep, to do anything. That’s what I want, sir.” (II-M-15-TR-C)
Theme 3: prosthesis component quality	“Just that the fingers used to remove [fall] quickly… and the nails… and [it is] peeling. …” (II-M-18-TR-T)
	“It [ideal prosthesis] should not be peeling… Because this one is also peeling. [It should be] like… normal… it didn’t use to peel.” (II-M-20-TH-C)
	“And also, the [prosthesis] color changes too soon, when I was getting dark… the thing is the color… changed not to the same color of this hand [sound limb].” (PF-M-16-TH-S)
	“I hope it [ideal prosthesis] will be waterproof.” (II-M-20-TH-C)
	“I wish this part was like… palm side, so I’ll be able to pour water on it.” (OF-F-18-TH-T)
Theme 4: physical comfort as key to prosthesis acceptance	
Inadequate fitting	“It’s [current prosthesis/socket] not comfortable because it’s tight.” (II-F-14-TH-T)
	IR: “Is it [current prosthesis/socket] tight?” PT: “Yes, it is tight now.” (II-M-18-TR-T)
	“I don’t like the way that they tight it [current prosthesis].” (TT-F-6-TH-T)
Excessive weight	“Sir, the only thing is that sometimes it’s [current prosthesis] kind of heavy and always uncomfortable sometimes. That’s the only thing. …I would love to make it lighter so that it will be comfortable for me to wear it around.” (OF-F-18-TR-T)
	“It [ideal prosthesis] should be light; it shouldn’t be heavy at all.” (PF-M-14-TH-T)
Ineffective suspension	“If I am going out, it [current prosthesis] can just drop down… If I am going out, it would just drop down on the floor.” (II-M-15-TR-C)
Insufficient prosthesis ventilation	“I don’t wear for days. I wear it [current prosthesis] for like some hours then I remove it… because of like… it gives me skin reaction… You should be able to wear it for days… without removing it because I do like sleep-overs at some friends’ house. So, I can’t… remove it.” (II-M-20-TH-C)
	“Yes, exactly. Recently when I wore it [current prosthesis]… my stump was red, so I had to leave it for a few days. I could not wear it for a few days.” (OF-F-18-TR-T)
	“It [current prosthesis] makes my hands [stump] sweat.” (II-M-18-TR-T)
Theme 5: anthropomorphic prostheses as tools for self-concept improvement and social integration	
Anthropomorphic appearance	“I like it [current prosthesis] because when I go out, I feel confident… when going out… Because people don’t know I don’t have an arm.” (OF-F-18-TH-T)
	“I want the one [ideal prosthesis] that looks like normal.” (PF-M-10-TH-T)
	“I want to see fingers… with nails… I want to see thumbs. I’ll use it [ideal prosthesis] to make… if I snap pictures, to make me look like as if the hand wasn’t cut.” (PF-F-8-TR-S)
Prosthesis color	“[I like that] it [current prosthesis] almost looks like my color.” (PF-F-16-TR-C)
	“[I want it (ideal prosthesis) to be] more like skin, a color that would match my skin.” (II-M-18-TR-T)
	“The color should be similar to the other hand.” (PF-M-16-TH-S)

Abbreviations: C, congenital; F, female; II, individual interview; IR, interviewer; M, male; OF, online focus group; PF, in-person focus group; PT, participant; S, sickness; T, trauma; TH, transhumeral; TR, transradial; ULD, upper limb difference.

^a^ Representative quote highlighting the necessity to acquire the capacity to perform coordinated bimanual tasks.

**Table 4.** Overarching Theme 2: Psychosocial Implications

Theme	Quotation (data collection method–sex [M/F]–age [years]–ULD level–ULD etiology)
Theme 6: self-perception	“It [current prosthesis] makes me complete.” (II-M-19-TH-T)
	“It [current prosthesis] serves as a form of completion… [When] they see you without it, I can feel people looking at it. With it, I feel good, people do not notice I miss an arm.” (PF-M-16-TH-S)
	“I like it [current prosthesis] because when I go out, I feel confident… because people don’t know I don’t have an arm.” (OF-F-18-TH-T)
	“I like it [current prosthesis] because it helps me build my confidence: whenever I go out, people hardly notice that’s flat [amputated]. It boosts my confidence.” (OF-F-18-TR-T)
	IR: “How would it [ideal prosthesis] make you feel?” PT: “Normal.” IR: “[It (ideal prosthesis) will make you feel normal…] So you don’t think you are normal?” PT: “No.” (II-F-12-TH-T)
	“I will be so grateful. I’ll be happy. I’ll be excited… like if I’m complete.” (II-F-18-TH-T)
	“I will feel like I can do anything I want to… it gives… freedom.” (II-M-20-TH-C)
	“I’ll feel like I have my other hand/I want if I wear it [ideal prosthesis], it makes me feel like I have… I have my second hand.” (II-M-18-TR-T)
Theme 7: peers’ and strangers’ attitudes	“When I go out, the bad thing is also… People start staring and say… ‘What’s this girl wearing?’ when people notice. That’s the thing.” (OF-F-18-TR-T)
	IR: “How do you feel When you wear it [current prosthesis] to church?” PT: “Somehow… like… the way people [look at me]… I don’t like.” (II-F-12-TH-T)
	“Also, most people don’t shake with the left… that can also [create problems].” (PF-M-16-TH-S)
	“… but sometimes if people want to shake me, I can’t.” (II-M-18-TH-T)

Abbreviations: C, congenital; F, female; II, individual interview; IR, interviewer; M, male; OF, online focus group; PF, in-person focus group; PT, participant; S, sickness; T, trauma; TH, transhumeral; TR, transradial; ULD, upper limb difference.

#### Functional and Mechanical Characteristics of an Ideal ULP

##### Functionality as a Prerequisite

Functional benefit was the most salient aspect of a ULP expressed by participants. For participants with traumatic limb loss, this emphasis reflected comparison with prior abilities, whereas for children and adolescents with ULDs due to congenital limb loss, it was associated with a sense of completeness and effectiveness in bimanual tasks. The identified subthemes were (1) frustrating passive prosthesis and (2) required functionalities for improved autonomy.

1. Most participants expressed frustration and dissatisfaction with their passive prostheses, highlighting minimal movement and limited utility despite acceptable cosmesis. As 2 participants noted, “I don’t like anything… because it’s not functioning,” and “I don’t use it to do anything… it gives me fitting [for aesthetics].”
2. Participants identified essential tasks an ideal active ULP (IAULP) should enable, grouped into 4 domains: (1) self-care, (2) household chores, (3) interpersonal interactions, and (4) other functional abilities. (1) children and adolescents with ULDs wished for improved autonomy in self-care (eg, hygiene) and daily living activities using an IAULP, which was currently unachievable, as it would result in lesser dependence on caregivers. (2) Children and adolescents with ULDs emphasized the necessity to perform household chores, marking enhanced autonomy and contribution to family life. In particular, the inability to hand wash clothes emerged as a critical barrier to independence across all ages. (3) The capacity to (re)engage in peer play and physical social interactions was also highly valued, especially by male participants, using terms such as *hug* and *holding/shaking hands* to signify comradery. (4) Children and adolescents with ULDs also shared individualized goals (eg, swimming) or vocational activities (eg, carpentry) that were currently inaccessible but that an IAULP should facilitate.

Collectively, these actions underscored the participants’ needs of coordinated bimanual function.

##### Ideal Active Prosthetic Design Specifications

Participants emphasized that achieving these functional goals requires an active, actuatable prosthesis with movable joints and fingers and controllable components. They described these functional characteristics using terms such as *flexible*, *movable*, *control*, and *functional*, mimicking “what the other hand can do.” Hence, children and adolescents with ULDs need an active ULP (AULP) providing utility through functional movement. Preferences regarding TDs reflected a balance between anthropomorphic, hand-shaped TDs for convenience of daily use and activity-specific TDs for versatility in specialized tasks.

##### Prosthesis Component Quality

Participants reported mechanical failures and wear leading to psychosocial discomfort, reduced social engagement, and limited use. Furthermore, waterproof and immersion-resistant ULP designs were frequently requested, reflecting daily activity needs (eg, household chores and hand washing clothes) and leisure interests (eg, swimming). The components’ reliability, durability, and material quality are critical for sustained use. This highlights the interconnection between prosthesis design and manufacturing, which requires alignment between anticipated functionality, component availability, material selection, and user needs.

##### Physical Comfort as Key to Prosthesis Acceptance

Comfort emerged as a central determinant of acceptance; a functionally adequate AULP could be rejected if it were uncomfortable. Participants indicated key contributors to discomfort to address for an IAULP: (1) inadequate fitting, (2) excessive weight, (3) ineffective suspension, and (4) insufficient prosthesis ventilation.

Children and adolescents with ULDs predominantly reported (1) poor socket fitting—due to inappropriate initial sizing or growth—causing high internal pressure on residual limbs as well as pain, which led then to restricting use to social situations. (2) Excessive weight induced painful wear and further limited acceptance. (3) Some participants described how defective suspension systems resulted in frequent unexpected prosthesis detachment, attracting unwanted attention, increasing stigma, and reducing social confidence. Proactive attempts to improve fastening were unsatisfactory. (4) Some children and adolescents with ULDs reported dermatologic lesions due to inadequate socket ventilation, causing temporary discontinuation of the use of ULPs with functional limitations.

##### Anthropomorphic Prostheses as Tools for Self-Concept Improvement and Social Integration

The final essential requirement of a physical IAULP was its anthropomorphic appearance—cosmesis was identified as crucial. All participants emphasized the necessity of wearing ULPs that “look like [their] normal arm” to blend in socially, answering participants’ needs for apparent “normality.” Two subthemes emerged: (1) anthropomorphic appearance and (2) prosthesis color. (1) Children and adolescents with ULDs described anthropomorphic appearance as critical for self-esteem, enhanced use, and social integration. Participants expressed a strong desire for prostheses resembling a “normal arm,” particularly regarding hand shape. (2) Similarly, nearly all children and adolescents with ULDs preferred prosthesis colors that match skin tone, using terms such as *my normal skin*, *chocolate*, and *brown*. Three participants favored alternative colors, suggesting openness to nonanthropomorphic designs.

#### Psychosocial Implications

Findings showed that ULP use was closely intertwined with self-concept and social interaction. Children and adolescents with ULDs provided valuable insights into their self-perception and the effect of others’ attitudes.

##### Self-Perception

Participants described how prosthesis use shaped feelings of confidence, normality, and completion, mediated largely by strangers’ reactions to their ULP anthropomorphism. Disruptions to this perceived normality induced uneasiness and influenced wear. Children and adolescents with ULDs, particularly participants with traumatic ULDs, also explained how they would feel “normal again” if wearing an anthropomorphic IAULD.

##### Peers’ and Strangers’ Attitudes

Participants shared how social stigma and discrimination due to their ULD or ULP, as well as cultural expectations (eg, right-hand handshakes), contributed to discomfort and social withdrawal, with some declaring it led to “not having/making friends.” For instance, inability to greet with right-hand handshakes, due to passive prostheses, led to adults’ adverse reactions, causing distress and shame. An IAULP capable of facilitating culturally normative interactions, such as handshaking, was therefore highly desired. The comments of children and adolescents with ULDs reflected self-consciousness about their conditions, functional limitations, and the negative social image associated with their disability.

### Summary of Findings

In summary, participants’ accounts highlighted that successful pediatric ULP development in LMICs requires clear task-based objectives and designs that integrate function, comfort, durability, and anthropomorphism, while supporting self-acceptance and social participation. No meaningful differences were observed by sex, age, limb difference etiology, or data collection method.

## Discussion

This study provides novel insights into the perceptions, needs, and expectations of children and adolescents with ULDs in a LMIC context, a population largely underrepresented in current literature. Our findings highlight how sociocultural and environmental factors shape ULP use and acceptance, underscoring the need for context-specific management strategies.

### Participant Characteristics and Priorities

Our study population diverged from cohorts in HICs. Participants predominantly experienced traumatic, transhumeral limb loss, consistent with LMIC epidemiology, whereas HIC cohorts experienced mainly congenital and transradial limb loss.^6,8,19,20,21,31,32^ Although the incidence of congenital ULDs in HICs ranges from 5.6 to 27.2 per 10 000 live births, the prevalence in LMICs remains unquantified due to absent registries.^19,20,33,34,35,36,37^ Second, unlike children and adolescents with ULDs in HICs—who often have access to active prostheses—^3,4^ all participants used passive devices, regardless of age. These differences likely explain the emphasis on functional restoration in our cohort, compared with the prioritization of cosmesis for children and adolescents with ULDs in HICs who have near-typical physical functioning without prostheses.^6,7,8,9,10,11^ Despite differing contexts, both groups identified common factors associated with prosthesis rejection: weight, comfort, durability, and appearance.^8,38^ Also, contrasting with cohorts in HICs, prosthesis abandonment was uncommon among participants in our study, likely reflecting strong social pressures to continue to wear the prosthesis despite dissatisfaction.^5,8^ Further comparison with adults in LMICs with ULDs highlighted similar comments regarding comfort issues and the social role of ULPs, affecting device acceptance.^24,39^

### Prosthesis Function and Access

Participants consistently identified the absence of active functionality as a major limitation of their passive prostheses. ULP active utility is critical for prosthesis acceptance and may affect the development of children and adolescents with ULDs.^1,2,4,5,7,40^ However, although early fitting of an active ULP (body-powered, myoelectric) is recommended,^2,3,4,40,41^ their high cost and the need for frequent replacements during growth substantially limit their access for children and adolescents with ULDs in LMICs,^1,4,11,17,18^ leaving many children reliant on passive prostheses until adulthood or without prosthetic care. Furthermore, access to activity-specific TDs—which were proven to enhance independence, self-concept, and ULP acceptance^1,3,4,12,15,40,41^—is similarly constrained,^15,17,18,23^ hindering recreational, educational, and professional opportunities.

### Play

Participants stressed the need for an IAULP enabling play with peers. This is critical, as social stigma, bullying, isolation, impaired self-image, and inherent physical limitations hinder their participation, while play is essential for children’s development.^12,42,43,44^ Therefore, development of a robust, context-specific AULP should account for the ability of children and adolescents with ULDs in LMICs to engage in play.^1,18^

### Comfort and Design Constraints

Comfort is a critical determinant of prosthesis use.^4,5,38,45,46,47^ Inadequate socket fitting and excessive weight can cause pain, dermatologic lesions, and inefficient device control, particularly for children with proximal impairments, contributing to rejection.^1,4,12,17,21,31,38,40,45,46,47,48^ Lightweight, customized pediatric designs may mitigate these challenges, and adjustable sockets support adaptation to growth while facilitating replacement strategy, limiting social and educational repercussions of nonuse periods.^17,18,49^ In addition, use (eg, childhood play) and environmental constraints—heat, humidity, dust, water exposure, and limited maintenance capacity^1,16,17,18^—further necessitate the development of robust, customized, lightweight, weather-resistant, and affordable ULP designs, favoring nonelectronic solutions.^17,18^

### Cosmesis

Cosmesis also plays a vital role in ULP acceptance.^1,5,38,46,47^ However, while some experts ranked anthropomorphic ULP shape over color,^46^ participants valued both aspects as essential for reducing stigma and improving self-perception and social engagement. Nevertheless, studies reported that children and adolescents with ULDs in HICs described hand-shaped generic AULPs as hindering functional tasks in comparison with nonanthropomorphic activity-specific TDs.^3,4,12,40,50^ Therefore, a user-centered design of pediatric ULP TDs is crucial in LMICs, as sociocultural load might adversely affect device acceptance,^17,18,51,52,53^ despite improved functionality.^1^

### Psychosocial Implications

Consistent with prior research,^1,2,3,4,53,54,55^ children and adolescents with ULDs frequently experienced reduced self-acceptance, impaired body image, low self-esteem, and social isolation due to their condition and prosthesis use, shaped by interactions with their social environment (family and peers) and stigma from the broader community.^1,17,52,56,57,58,59,60^ Drawing from the Goffman theory of stigma^61^—a socially constructed phenomenon, stigmatized children and adolescents with ULDs protect themselves by “managing their identity” through concealing (ULP use) the “discrediting” attribute (ULD) to appear normal or through trying to mitigate it during social interactions.^11,61^ This dynamic underlies the interconnection between ULP anthropomorphism (shape, color, and motion) and the sociocultural pressures shaping ULP acceptance. Despite this burden, psychosocial assessment is rarely integrated into care.^4,11,54^ Strengthening collaboration among health care professionals, schools, and families is essential to support mental health and inclusion.^4,11,12,40,52,53,54,55,56,57,59,60,62,63^

### Design Trade-Off Necessity

This discussion emphasizes the need for a balanced, pediatric user–centered design approach to ULPs for children and adolescents with ULDs in LMICs. Our findings highlight interrelated design requirements that must account for locally available technologies and aforementioned environmental constraints.^4,12,13,14,17,18^ Myoelectric ULPs provide advanced anthropomorphism but are costly, heavy, and fragile,^1,45,64^ whereas body-powered ULPs are more affordable and durable but often bulky and less comfortable.^1,13,45,64^ Activity-specific TDs enable specialized functions but require frequent changes,^4,12,14^ while anthropomorphic passive prostheses offer limited functional benefit.^1,13,45,64^ Structural barriers (access to rehabilitation services and importation of components) and environmental constraints further necessitate individualized solutions through shared decision-making and user education to promote satisfaction and sustained use.^1,4,11,13,14,17,18,65^

### Integrated Framework and Recommendations

Taken together, these findings support a holistic, biopsychosocial approach to pediatric ULP provision in LMICs. Analyzing results using the International Classification of Functioning, Disability and Health (ICF) provides a framework to identify key and contextual factors while facilitating comparison across populations^63,66,67,68^ and informing clinical, design, and policy interventions.^63,66,67,68^ Within the ICF framework, children and adolescents with ULDs in our study have a body structure impairment (forearm or upper arm) associated with substantial functional limitations, compounded by inadequate prostheses, with environmental and personal factors further influencing activities and participation. A proposed classification is presented in the eTable in Supplement 1.

We recommend coordinated, multistakeholder (patients, caregivers, clinicians, manufacturers, nongovernmental organizations, and policymakers) strategies, encompassing (1) systematic assessment of individual occupational and psychosocial needs through validated instruments and frameworks, (2) development of context-appropriate prosthetic solutions using locally available technologies, and (3) public policies to reduce stigma and promote inclusion.

### Limitations

This study has several limitations. Because TIF staff who moderated discussions and interviews were involved in prosthetic care, social desirability bias may have occurred. This was mitigated through promoting a nonjudgmental atmosphere through games, reassurance about the absence of repercussions, and team-based discussions of recorded potential bias. Telephone interviews limited nonverbal data capture and the use of illustrative material, and translation from Yoruba without formal validation may have introduced interpretation bias. Although the small sample size limits generalizability and although selection bias was possible because the participants who engaged in prosthetic care may have differed from unserved children, the rich contextual details provided in our study enhanced transferability.

## Conclusions

In this qualitative study, Nigerian youths with ULDs described unmet needs and expectations. The findings underscored the persistent disparities in pediatric prosthetic access between HICs and LMICs. Participants expressed the need for anthropomorphic, comfortable, and robust active ULPs that enable confident engagement in daily activities and occupations. Further research and the development of appropriate pediatric ULPs—along with public policies—within these sociocultural contexts are warranted.

## Notes

Supplement 1. **eMethods. **Questioning Guide **eTable. **Suggested ICF Applications to Study Results

Supplement 2.
Data Sharing Statement
