# Supplementary material for: Needs and Perspectives on Upper Limb Prostheses Among Children and Adolescents With Upper Limb Differences
Source: JAMA Netw Open. 2026 Jun 25;9(6):e2620122. doi: 10.1001/jamanetworkopen.2026.20122 (PMC13306300; doi:10.1001/jamanetworkopen.2026.20122)
Supplement: Supplement 1. — eMethods. Questioning Guide eTable. Suggested ICF Applications to Study Results [file jamanetwopen-e2620122-s001.pdf]

Supplementary Online Content

Wendo K, Chigbu C, Ekundayo O, et al. Needs and perspectives on upper limb prostheses among children and adolescents with upper limb differences. *JAMA Netw Open.* 2026;9(6):e2620122. doi:10.1001/jamanetworkopen.2026.20122

eMethods. Questioning Guide

eTable. Suggested ICF Applications to Study Results

This supplementary material has been provided by the authors to give readers additional information about their work.

© 2026 Wendo K et al. *JAMA Network Open.*

eMethods. Questioning Guide

1) **Opening questions (Focus Group)**

Tell us your name and from which city you come from.

Tell us your first name and what is your favorite dish.

1) **Opening questions (Interviews)**

Greetings and making them feel comfortable.

Tell us about is your favorite dish.

2) **Beginning – 1st stage questions (both interview and focus group) - Important part**

I would like you to think about your prosthesis.

What are the things you like about it?

What are the things you don't like about it?

What is good or bad about the way they look?

What is good or bad about the things you can do with them?

What is good or bad about the way they feel?

Are they comfortable? What does or doesn't help them to be comfortable?

(For older champions (participants), asking if they had other prostheses before. If so, asking about the things they liked and didn't like about, which one they preferred and why).

(For older champions, asking what it would need to be helpful during their primary school/high-school time).

3) **3rd stage questions (both interview and focus group) – Important part**

I would like you to imagine that I have superpowers and I can make your ideal/best prosthesis, just for me:

Tell me:

What would it need to look like? For you to like it.

- ⇨ The color.
- ⇨ Special/specific features.
- ⇨ Feel/texture of the surface. (when you touch it or when it touches your skin)

(Maybe drawing)

What type of activities would it need to help you to do?

- ⇨ First, at home.
- ⇨ Second, at school.

- ⇨ Third, with friends.

(Maybe drawing)

What would you like it to have at the end of it to help you? (showing the end of a prosthesis)

- ⇨ A hand (that can move or not).
- ⇨ A tool that can do different things.
- ⇨ Something else.

(Maybe drawing)

How would you feel about it?

How would it feel when you are wearing it?

What would other people think about it? (Family, friends…)

What would help you to use the prosthesis more often?

(For older champions, asking what it would need to be helpful during their primary school/high-school time).

4) Final stage

Interview

Is there anything else that you would like to tell me about? Something we have already not talked about your ideal prosthesis or what could be improved in prostheses to help you.

Focus group

Is there anything else that anyone would like to share about your ideal prosthesis? Something we have already not talked about.

© 2026 Wendo K et al. *JAMA Network Open.*

Illustrating photographs of different terminal device options, which were used during focus groups:

- Anthropomorphic terminal device

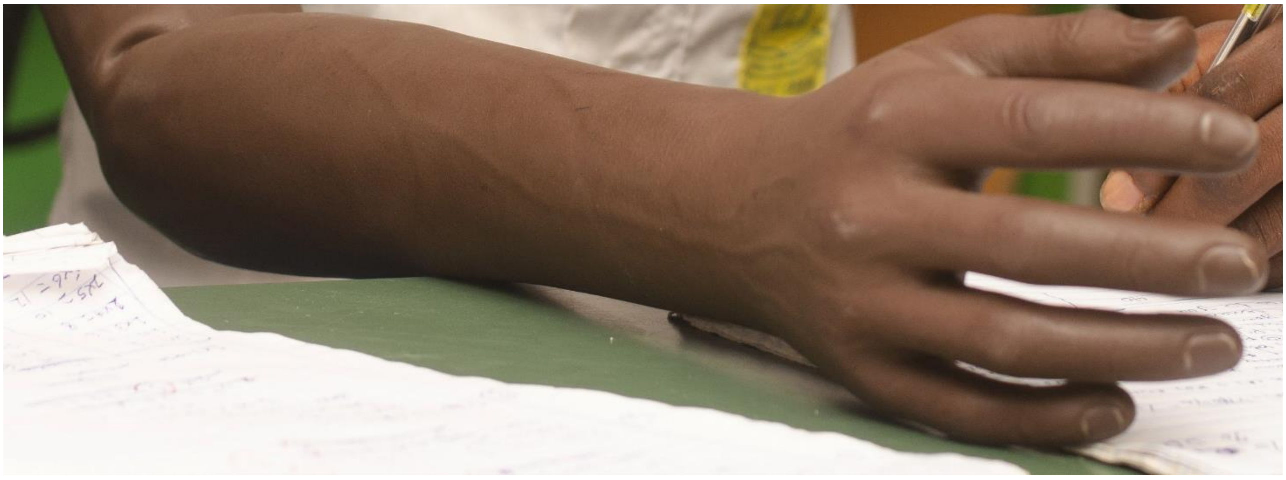

- Non-anthropomorphic activity-specific terminal device

Our team was not the originator of the device presented on the second photograph and did not have permission to publish the image. The terminal device consisted of a pen holder attached perpendicularly to a forearm outer socket, allowing the user to write.

© 2026 Wendo K et al. *JAMA Network Open.*

eTable. Suggested ICF Applications to Study Results

(Sub-)themes	Body Structures	Body Functions	Activities & Participation	Environmental Factors
	s7300 Structure of upper arm<br>s7301 Structure of forearm<br><br>*Always applicable*	b710 Mobility of joint functions<br>b720 Mobility of bone functions<br>b730 Muscle power functions<br><br>*Always applicable*		e555 Services, systems and policies related to associations<br>e560 Media services, systems and policies<br>e570 Social security services, systems and policies<br>e580 Health services, systems and policies<br>e585 Education and training services, systems and policies<br><br>*Always applicable*
**Functional and mechanical characteristics of an ideal upper limb prosthesis**				
1. *Functionality as prerequisite*			d430 Lifting and carrying objects<br>d440 Fine hand use<br>d445 Hand and arm use	
• Frustrating passive prosthesis		b180 Experience of self and time functions<br>b1800 Experience of self<br>b1801 Body image	d210 Undertaking a single task<br>d220 Undertaking multiple tasks	e115 Products and technology for personal use in daily living<br>e1151 Assistive products and technology for personal
• Required functionalities for improved autonomy				
○ *Self-care*			d510 Washing oneself<br>d520 Caring for body parts<br>d530 Toileting<br>d540 Dressing<br>d550 Eating<br>d560 Drinking	e310 Immediate family
○ *House chores*			d630 Preparing meals<br>d640 Doing housework<br>d650 Caring for household objects<br>d660 Assisting others	e310 Immediate family<br><br>e410 Individual attitudes of immediate family members
○ *Interpersonal interactions*			d720 Complex interpersonal interactions<br>d730 Relating with strangers<br>d740 Formal relationships<br><br>d910 Community life	e320 Friends<br>e325 Acquaintances, peers<br><br>e420 Individual attitudes of friends<br>e425 Individual attitudes of acquaintances

© 2026 Wendo K et al. *JAMA Network Open.*

eTable. Suggested ICF Applications to Study Results (Continued)

(Sub-)themes	Body Structures	Body Functions	Activities & Participation	Environmental Factors
○ *Other functional abilities*			d475 Driving<br><br>d820 School education<br><br>d910 Community life<br>d920 Recreation and leisure	e460 Societal attitudes
2. *Active Prosthetic design specifications*			d430 Lifting and carrying objects<br>d440 Fine hand use<br>d445 Hand and arm use<br><br>d640 Doing housework<br>d650 Caring for household objects<br><br>d720 Complex interpersonal interactions	e115 Products and technology for personal use in daily living<br>e1151 Assistive products and technology for personal use
3. *Prosthesis component quality*			d430 Lifting and carrying objects<br><br>d510 Washing oneself<br>d520 Caring for body<br><br>d920 Recreation and leisure	e115 Products and technology for personal use in daily living<br>e1151 Assistive products and technology for personal use<br>e225 Climate<br>e2250 Temperature<br>e2251 Humidity
4. *Comfort as key to prosthesis acceptance*				
• Inadequate fitting		b298 Sensory functions and pain, other specified<br><br>b840 Sensation related to the skin		e115 Products and technology for personal use in daily living<br>e1151 Assistive products and technology for personal use
• Excessive weight		b298 Sensory functions and pain, other specified<br><br>b840 Sensation related to the skin		e115 Products and technology for personal use in daily living<br>e1151 Assistive products and technology for personal use
• Ineffective suspension		b180 Experience of self and time functions<br>b1800 Experience of self<br>b1801 Body image		e115 Products and technology for personal use in daily living<br>e1151 Assistive products and technology for personal use<br><br>e345 Strangers<br>e445 Individual attitudes of strangers

© 2026 Wendo K et al. *JAMA Network Open.*

eTable. Suggested ICF Applications to Study Results (Continued)

(Sub-)themes	Body Structures	Body Functions	Activities & Participation	Environmental Factors
• Insufficient prosthesis ventilation		b840 Sensation related to the skin		e115 Products and technology for personal use in daily living<br>e1151 Assistive products and technology for personal use<br>e225 Climate<br>e2250 Temperature<br>e2251 Humidity
5. Anthropomorphic prostheses as tools for self-concept improvement and social integration				
• Anthropomorphic appearance		b180 Experience of self and time functions<br>b1800 Experience of self<br>b1801 Body image	d720 Complex interpersonal interactions<br>d730 Relating with strangers<br>d740 Formal relationships<br><br>d910 Community life	e310 Immediate family<br>e320 Friends<br>e325 Acquaintances, peers<br>e330 People in positions of authority<br>e345 Strangers<br><br>e410 Individual attitudes of immediate family members<br>e415 Individual attitudes of extended family members<br>e420 Individual attitudes of friends<br>e425 Individual attitudes of acquaintances<br>e430 Individual attitudes of people in authority<br>e445 Individual attitudes of strangers<br>e460 Societal attitudes
• Prosthesis color		b180 Experience of self and time functions<br>b1800 Experience of self<br>b1801 Body image	d720 Complex interpersonal interactions<br>d730 Relating with strangers<br>d740 Formal relationships<br><br>d910 Community life	e310 Immediate family<br>e320 Friends<br>e325 Acquaintances, peers<br>e330 People in positions of authority<br>e345 Strangers<br><br>e410 Individual attitudes of immediate family members<br>e415 Individual attitudes of extended family members<br>e420 Individual attitudes of friends<br>e425 Individual attitudes of acquaintances<br>e430 Individual attitudes of people in authority<br>e445 Individual attitudes of strangers<br>e460 Societal attitudes

© 2026 Wendo K et al. *JAMA Network Open.*

eTable. Suggested ICF Applications to Study Results (Continued)

(Sub-)themes	Body Structures	Body Functions	Activities & Participation	Environmental Factors
**Psychosocial implications**				
6. Self-perception		b180 Experience of self and time functions<br>b1800 Experience of self<br>b1801 Body image	d720 Complex interpersonal interactions<br>d730 Relating with strangers<br>d740 Formal relationships<br><br>d910 Community life	e310 Immediate family<br>e320 Friends<br>e325 Acquaintances, peers<br>e330 People in positions of authority<br>e345 Strangers<br>e355 Health professionals<br><br>e410 Individual attitudes of immediate family members<br>e415 Individual attitudes of extended family members<br>e420 Individual attitudes of friends<br>e425 Individual attitudes of acquaintances<br>e430 Individual attitudes of people in authority<br>e445 Individual attitudes of strangers<br>e450 Individual attitudes of health professionals<br>e460 Societal attitudes
7. Peers‘ and strangers’ attitudes		b180 Experience of self and time functions<br>b1800 Experience of self<br>b1801 Body image	d720 Complex interpersonal interactions<br>d730 Relating with strangers<br>d740 Formal relationships<br><br>d910 Community life	e310 Immediate family<br>e320 Friends<br>e325 Acquaintances, peers<br>e330 People in positions of authority<br>e345 Strangers<br>e355 Health professionals<br><br>e410 Individual attitudes of immediate family members<br>e415 Individual attitudes of extended family members<br>e420 Individual attitudes of friends<br>e425 Individual attitudes of acquaintances<br>e430 Individual attitudes of people in authority<br>e445 Individual attitudes of strangers<br>e450 Individual attitudes of health professionals<br>e460 Societal attitudes

Abbreviations: ICF, International Classification of Functioning, Disability and Health

© 2026 Wendo K et al. *JAMA Network Open.*
