# Supplementary material for: Needs and Perspectives on Upper Limb Prostheses Among Children and Adolescents With Upper Limb Differences
Source: JAMA Netw Open. 2026 Jun 25;9(6):e2620122. doi: 10.1001/jamanetworkopen.2026.20122 (PMC13306300; doi:10.1001/jamanetworkopen.2026.20122)
Supplement: Supplement 2. — Data Sharing Statement [file jamanetwopen-e2620122-s002.pdf]

Data Sharing Statement

Wendo. Needs and Perspectives on Upper Limb Prostheses Among Children and Adolescents With Upper Limb Differences. *JAMA Netw Open*. Published June 25, 2026.
doi:10.1001/jamanetworkopen.2026.20122

Data
Data available: Yes
Data types: Deidentified participant data
How to access data: Data requests can be emailed to corresponding author at kevin.wendo@uclouvain.be
When available: With publication

Supporting Documents
Document types: Other (please specify)
Additional Information: Questioning guide, Informed consent form, Assent form, Sociodemographic data collection form.
How to access documents: Data requests can be emailed to corresponding author at kevin.wendo@uclouvain.be
When available: With publication

Additional Information
Who can access the data: Due to the relatively small sample sizes intrinsic to qualitative research, a small risk exists for participant identification even following rigorous de-identification procedures. Given this risk, our research team does not share entire raw data sets upfront to all-comers. We are enthusiastic about sharing de-identified data on a case-by-case basis to researchers under a data-sharing agreement in the setting of an IRB approved research protocol and explicit assurance that data will be reviewed and analyzed exclusively for research purposes without identification of individual participants.
Types of analyses: Any purpose.
Mechanisms of data availability: We are enthusiastic about sharing de-identified data on a case-by-case basis to researchers under a data-sharing agreement in the setting of an IRB approved research protocol and explicit assurance that data will be reviewed and analyzed exclusively for research purposes without identification of individual participants.
Any additional restrictions: N/A.
